# INTRABEAM intraoperative radiotherapy combined with portal vein infusion chemotherapy for treating hepatocellular carcinoma with portal vein tumor thrombus

**DOI:** 10.1186/s12893-020-00836-2

**Published:** 2020-08-01

**Authors:** Xiaodong Song, Yong He, Huihong Liang, Menling Han, Zili Shao

**Affiliations:** Department of Hepatobiliary Surgery, The Second Affiliated Hospital of Guangzhou Medical University, No. 250, Changgang Road, Guangzhou, 510260 P. R. China

**Keywords:** Portal vein tumor thrombus, Hepatocellular carcinoma, Intraoperative radiotherapy, Portal vein infusion chemotherapy

## Abstract

**Background:**

Portal vein tumor thrombus (PVTT) is common in hepatocellular carcinoma (HCC). Recent studies indicate that more aggressive treatments, including surgical resection or locoregional treatment, may benefit selected HCC patients with PVTT. External radiation therapy and infusion chemotherapy were found to achieve good outcomes; however, the use of low-energy x-ray radiation system (INTRABEAM), intraoperative radiation therapy, and portal vein infusion chemotherapy for PVTT has not been reported.

**Case summary:**

We present a case of HCC with PVTT. The patient underwent hemihepatectomy and thrombectomy along with intraoperative radiotherapy (IORT) using a portable INTRABEAM radiation system. Subsequently, to treat PVTT, portal vein infusion chemotherapy with FOLFOX (leucovorin [Folinic acid], fluorouracil, and oxaliplatin) regimen was administered. There were no obvious post-operative complications. After 20 months follow-up period, no obvious tumor recurrence had been observed, and PVTT gradually disappeared completely.

**Conclusions:**

IORT using the INTRABEAM radiation system combined with portal vein infusion chemotherapy is promising for select patients with PVTT.

## Background

Hepatocellular carcinoma (HCC) is the fourth most common malignancy and the third leading cause of tumor-related deaths in China. It poses a significant threat to the life and health of people [1]. Patients with HCC can develop portal vein tumor thrombus (PVTT) due to direct extension or by intravascular metastases. The incidence of PVTT in HCC ranges from 10 to 60% [2], which is believed as one of the most reliable prognostic factors for the survival of HCC patients [3]. The median survival time in untreated HCC patients without PVTT is 24.4 months; however, in patients with PVTT, it is 2.7 months [4] and thus, indicates a necessity for curing and controlling PVTT [5]. Currently, there is no consensus on how to best treat HCC patients with PVTT, and more studies indicate that aggressive treatments, including surgical resection or locoregional treatment, may benefit select HCC patients with PVTT [6, 7]. However, post-operative liver tumor recurrence rate was high (56.9%) and had a high PVTT recurrence rate in the short-term [8, 9]. Thus, effective and continuous treatment methods for PVTT are required. In recent years, proton beam radiotherapy has been used in PVTT patients, and has appeared to significantly improve survival and local control [10]. Cisplatin-based hepatic arterial infusion chemotherapy has been widely employed for HCC patients with PVTT [11, 12]. It is believed that chemotherapeutic agents were directly delivered into the tumor-feeding arteries and minimized systemic toxic effects producing a significantly higher response rate (22 to 48%) [13–17]. Yet, portal vein infusion chemotherapy was not reported.

We employed intraoperative radiotherapy (IORT) using the INTRABEAM system combined with portal vein chemotherapy to HCC with PVTT. We believe that it can treat PVTT definitively, decrease the liver tumor and PVTT recurrence rate, and improve the survival of patients.

## Case presentation

A 73-year-old Chinese man was diagnosed with a hepatic mass for two months and suffered a one-month history of right upper quadrant pain. The patient had not used any medications before being hospitalized.

After the patient was hospitalized, physical examination did not find any apparent abnormalities. The patient’s neurological status was determined to be normal. The patient’s family history was non-contributory. The patient had never consumed alcohol. He had a 20-year history of smoking, but the number of cigarettes smoked per day was unknown. The patient was a retiree, and he had been an office staff 20 years before.

**Fig. 1:**
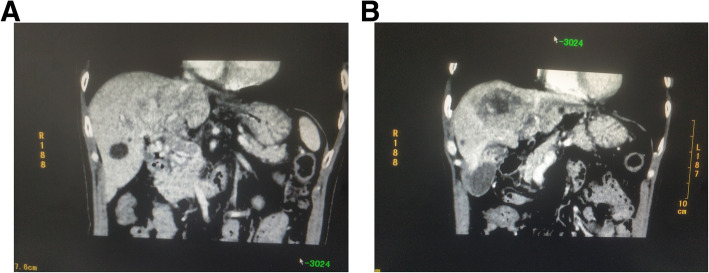
Representative computed tomography scan images before the operation. **a**. White circle showing the portal vein tumor thrombus, and that the portal vein main trunk was invaded; this was classified as grade VP4. **b**: White circle denoting the tumor located in the liver section IV; the approximate diameter is 5 cm. The tumor showed enhancement in the arterial phase and no enhancement in the portal and delayed phases

Laboratory blood tests (Table 1) were administrated and revealed a high level of the AFP tumor marker (337.83 μg/L). There were no signs of Hepatitis virus infection. The patient was found to have Child-Pugh Class A liver function, and abdominal computed tomography with enhancement and vascular reconstruction revealed that there was a lesion in section IV of the liver, with an approximate diameter of 5 cm. In addition, the tumor demonstrated enhancement in the arterial phase and no enhancement in the portal and delayed phases. There was a portal vein filling defect involving the main portal vein trunk, left portal vein, and right portal vein (Fig. 1). The patient was diagnosed with HCC with PVTT, which was accompanied by a grade VP4 portal vein invasion.

**Table 1:** Laboratory data on presentation

Variable	Values
Hemoglobin (g/dl)	11.7
Hematocrit (%)	38.4
White cell count (per mm^3^)	5500
Differential count (%)	
Neutrophils	56.5
Lymphocytes	26.0
Platelet count (per mm^3^)	163,000
Red cell count (per mm^3^)	3,700,000
Mean corpuscular volume (fl)	96
Sodium (mmol/liter)	142.7
Potassium (mmol/liter)	2.72
Chloride (mmol/liter)	107.1
Calcium (mg/dl)	2.21
Glucose (mmol/liter)	4.82
Urea nitrogen (mmol/liter)	3.29
Creatinine (umol/liter)	83.3
Protein total (g/dl)	6.29
Albumin (g/liter)	35.5
Alanine aminotransferase (U/liter)	51
Aspartate aminotransferase (U/liter)	96
Bilirubin total (umol/liter)	27.3
Lactate dehydrogenase (U/liter)	188
Urine analysis	Normal
Serology for toxoplasmosis, herpes virus, CMV, EBV, rubella	Negative
AFP (ug/liter)	337.83

**Fig. 2:**
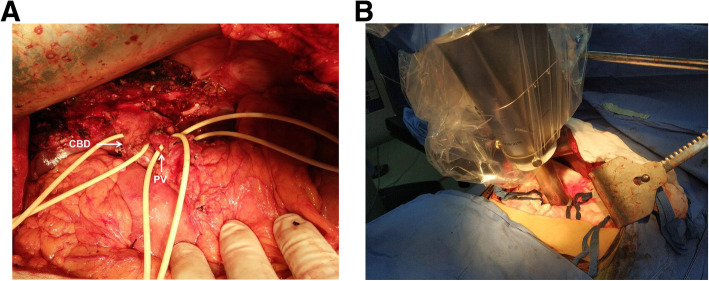
IORT using the INTRABEAM radiation system. **a**. White arrows indicating the anatomical structure of the first hilum. (CBD: Common bile duct. PT: portal vein). Macroscopic portal vein tumor thrombus was removed after opening the trunk of portal vein. **b** After the sutured portal vein incision, Intraoperative radiation therapy was performed using the INTRABEAM radiation system. The plat source applicator was placed close to the portal vein, and the surrounding bowel and organs were protected using two surgical gauze

**Fig. 3:**
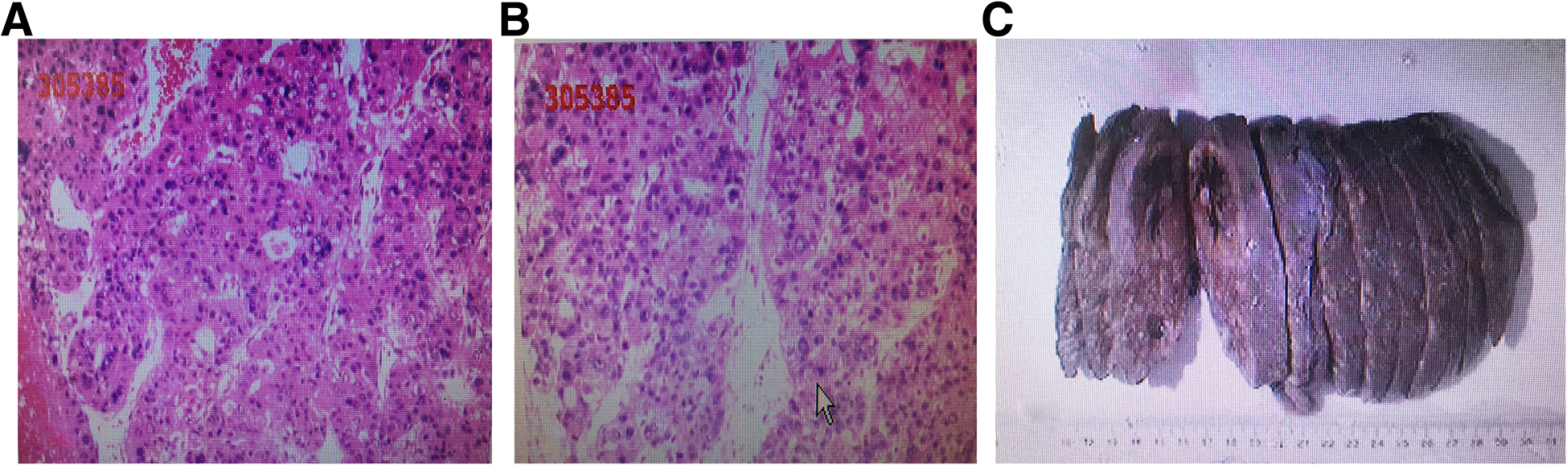
H&E stained tumor pathological slices. **a**: The HE-stained slices of liver tumor tissue. **b**: The HE-stained slices of portal vein thrombus. **c**: The resected tumor sample

Six days after admission, the patient underwent a left hemihepatectomy and thrombectomy accompanied with IORT. A subcostal incision was employed. After exposing the abdominal cavity, it was determined that the main lesion located in segment IV measured approximately 6 cm × 5 cm, and several satellite nodules were located in segment II and III. Hepatic inflow occlusion was performed using the Pringle’s maneuver. A liver resection was combined with the extraction of PVTT by opening the trunk of the portal vein. Any small PVTT in the tiny branches was suctioned. Then the portal vein incision was sutured. For radiological protection, several layers of surgical gauze were used to insulate nearby organs, while only exposing the surface of the trunk of the portal vein. A 4 cm flatbed source applicator of an INTRABEAM system was moved into the surgical field, and placed close to the portal vein (Fig. 2). After all personnel had left the operating room, radiotherapy was initiated using the following parameters: a dose of 15 Gy, an irradiation time of 22 mins, 30 s, an acceleration voltage of 50 kV, and an acceleration current of 40 μA. Afterwards, a catheter was inserted into the right gastro-omental vein, the infusion port was implanted in subcutaneous tissue for the post-operative portal vein infusion chemotherapy. The abdominal cavity was closed after confirming that there was no bleeding or biliary leakage. The total intraoperative blood loss was 200 mL. The resected tumor is shown in Fig. 3. A HE-stained pathological exam proved the tumor to be an HCC, grade II-III, with many satellite nodules and a microangiocarcinoma thrombus. The portal vein thrombus was shown to be a cancerous node.

The patient’s gastric tube was removed after gastroenteric function recovered on postoperative day two. There was less than 50 mL of fluid lost via the abdominal drainage tube on postoperative day eight, which was also removed. The patient was discharged on post-operative day nine. The blood biochemical indexes and the AFP level were found to be normal upon reexamination (Table 2). No surgery-related complications were observed. Due to the patient’s personal reason, they refused to take sorafinib. One month post-surgery, portal vein infusion chemotherapy of FOLFOX regimen was performed every three weeks. The following regimen was administrated via portal vein infusion port: oxaliplatin, 85 mg/m^2^, from hour 0 to 2 on day one; leucovorin, 400 mg/m^2^, from hour 2 to 3 on day one; fluorouracil, 400 mg/m^2^, bolus at hour 3; and 2400 mg/m^2^ over 46 h on days one and two. CT scanning and blood serum test were performed every two months. The patient had no side effect reactions in response to the chemotherapy. The post-operative AFP level was 6.15 μg/L.

**Table 2:** Laboratory data post operation

Variable	Values
Hemoglobin (g/dl)	10.4
Hematocrit (%)	38.0
White cell count (per mm^3^)	4.40
Differential count (%)	
Neutrophils	67.8
Lymphocytes	19.0
Platelet count (per mm^3^)	142,000
Red cell count (per mm^3^)	3,9000
Mean corpuscular volume (fl)	98
Sodium (mmol/liter)	142.9
Potassium (mmol/liter)	3.05
Chloride (mmol/liter)	106.7
Calcium (mg/dl)	2.37
Glucose (mmol/liter)	5.40
Urea nitrogen (mmol/liter)	5.93
Creatinine (umol/liter)	76.1
Protein total (g/dl)	7.49
Albumin (g/liter)	40.5
Alanine aminotransferase (U/liter)	43
Aspartate aminotransferase (U/liter)	53
Bilirubin total (umol/liter)	20.4
Lactate dehydrogenase (U/liter)	165
Urine analysis	Normal
AFP (ug/liter)	4.95

**Fig. 4:**
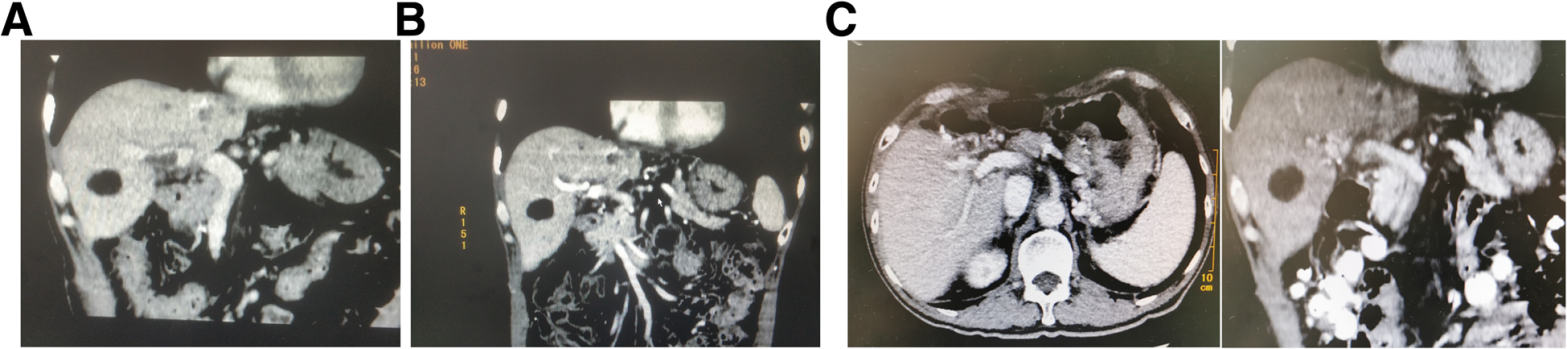
Representative computed tomography scan images after operation. **a**: A computed tomography scan images 2 months after the operation. Portal vein tumor thrombus reduced significantly. **b**: Computed tomography scan images 6 months after the operation. There were no portal vein filling defects, portal vein tumor thrombus was not overtly present in CT images. **c**: Computed tomography scan images 20 months after the operation. There was no tumor recurrence, portal vein tumor thrombus was not observable in the CT images

The patient had been followed every two months after surgery for 20 months, and no obvious tumor recurrence was observed. Moreover, the portal vein tumor thrombus was gradually reduced, and had disappeared after the sixth chemotherapy course (Fig. 4).

## Discussion and conclusion

Portal vein tumor thrombus is common in HCC. The presence of PVTT in patients with HCC is associated with poor prognoses [3, 4] due to impaired hepatic reserves, intrinsic aggressiveness of a tumor, and a high rate of developing complications related to portal hypertension. Portal vein invasion has been identified as a risk factor for recurrence and death after resection of HCCs [18].

Conventionally, HCC with PVTT is recognized as an advanced stage disease according to the Barcelona Clinic for Liver Cancer (BCLC) [19]. In western countries, the first-line treatment includes systemic therapy, such as sorafenib, while studies from China and Japan suggest that surgical treatment results in better outcomes. The treatment that we proposed in the present study is different from the current BCLC treatment algorithm, which is applied irrespective of the degree of portal vein invasion. However, since the population of HCC patients with PVTT is heterogeneous, some select patients with PVTT may benefit from aggressive treatment modalities as well [20].

Regarding the issue of surgical treatment of PVTT, preventing tumor and portal vein thrombus local recurrence after hepatectomy and thrombectomy are key points to be considered for treatment. To solve this problem, we suggest providing subsequent locoregional radiotherapy and chemotherapy to portal vein tumor thrombus after taking out the macroscopic tumor thrombus. The residual microscopic tumor thrombus could thus be eliminated through continuous subsequent treatment.

We presented a patient with HCC and PVTT who was successfully treated. The patient underwent a left hemihepatectomy and thrombectomy along with intra-operative radiotherapy (IORT) using an INTRABEAM radiation system. This low-energy portable IORT system decreases radiological side-effects. We employed it to eliminate any possible residual portal vein microscopic tumor thrombus after extracting the macroscopic PVTT by surgery. It has been reported that radiotherapy should be considered for HCC with PVTT for the purpose of improving local control, and potentially delaying the worsening of venous obstruction and liver failure [21]. Unlike previous research, which was based on external radiation therapy, our radiation therapy approach is based on IORT which acts directly on the treatment site. Intra-operative radiotherapy is believed to deliver a large radiation dose compare of separate radiation doses, while sparing the surrounding organs and tissues, and also shortening the overall treatment time [22]. In this case, we used INTRABEAM radiation system for IORT because it produces softer, shallower, and lower energy X-ways (30–50 kV) than traditional radiation systems do, such as Mobetron [21–26]. Thus, it can reduce various radioactive related adverse effects [27]. The INTRABEAM radiation system provides low-penetration depth of approximately 5 mm [21], and a relatively high-radiation dose on the surface of the radiation site. Consequently, we performed IORT with INTRABEAM radiation system to treat PVTT, meanwhile producing minor radioactive side reactions.

Furthermore, portal vein catheterization was administrated in the right gastro-omental vein for portal vein infusion chemotherapy post-surgery providing a forward systemic treatment to PVTT.

Hepatic arterial infusion chemotherapy consisting of leucovorin (Folinic acid), fluorouracil, and oxaliplatin (FOLFOX) was demonstrated to improve overall survival among patients with HCC and portal vein invasion while producing acceptable treatment-relative toxic effects [28]. As a locoregional treatment method, infusion chemotherapy for HCC patients with PVTT has shown a promise to be safe and effective.

Because of the anatomical features of portal vein thrombus, we believe that infusion chemotherapy by portal vein plays a more precise and direct role than arterial infusion chemotherapy to eliminate cancer thrombus. Vascular intervention treatment is relatively complex and risky when compared to portal vein catheterization. Portal vein catheterization during open surgery has its convenient advantages. Catheterization was administrated in the right gastro-omental vein, and the infusion port was implanted in the hypodermic for post-operative chemotherapy administration.

In this case, one month after the operation, portal vein infusion chemotherapy of FOLFOX regimen was performed every three weeks. The chemotherapy procedure went smoothly and the patient did not complain of any discomfort. Post-operative follow-up showed that the PVTT gradually reduced. Although the primary PVTT was clear, the left PVTT was present two months after the operation, while there was no portal vein filling defects after undergoing six chemotherapy courses, six months after the operation. These middle to long-term positive outcomes were considered to be attributable to the portal vein infusion chemotherapy. It proved that IORT and portal vein infusion chemotherapy is effective and safe, and played a comprehensive role in PVTT subsequent treatment.

Based on this case study of the HCC patient with PVTT, we conclude that using the low-energy INTRABEAM system for the IORT of these patients appears to be safe and can be applied for patients who have to undergo thrombectomy to eliminate suspicious tumor remnants. Furthermore, combined with subsequent portal vein infusion chemotherapy, a systematic treatment for patients with PVTT was followed. Our proposed method may benefit these patients. Large control studies are needed to validate our findings, which are promising for further future developments.

## Data Availability

All data generated or analyzed during this study are included in this published article and its supplementary information files.

## Abbreviations

IORT: Intraoperative radiation therapy
PVTT: Portal vein tumor thrombus
